# The Antioxidant Potential of Vitamins and Their Implication in Metabolic Abnormalities

**DOI:** 10.3390/nu16162740

**Published:** 2024-08-16

**Authors:** Panagiotis Theodosis-Nobelos, Eleni A. Rekka

**Affiliations:** 1 Department of Pharmacy, School of Health Sciences, Frederick University, Nicosia 1036, Cyprus; 2 Department of Pharmaceutical Chemistry, School of Pharmacy, Aristotelian University of Thessaloniki, 54124 Thessaloniki, Greece; rekka@pharm.auth.gr

**Keywords:** vitamins, oxidative stress, antioxidant activity, metabolic disorders, degenerative conditions

## Abstract

Vitamins are micronutrients necessary for the normal function of the body. Although each vitamin has different physicochemical properties and a specific role in maintaining life, they may also possess a common characteristic, i.e., antioxidant activity. Oxidative stress can harm all the main biological structures leading to protein, DNA and lipid oxidation, with concomitant impairment of the cell. It has been established that oxidative stress is implicated in several pathological conditions such as atherosclerosis, diabetes, obesity, inflammation and metabolic syndrome. In this review we investigate the influence of oxidative stress on the above conditions, examine the interrelation between oxidative stress and inflammation and point out the importance of vitamins in these processes, especially in oxidative load manipulation and metabolic abnormalities.

## 1. Introduction

**Figure 1:**
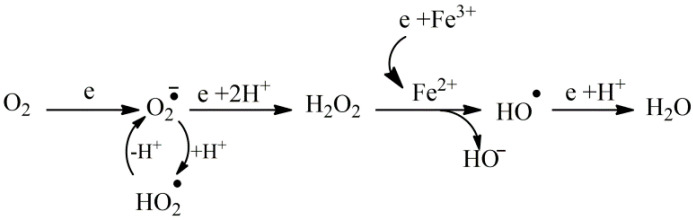
The one electron reduction process of oxygen. Molecular oxygen may be reduced to superoxide anion radical that according to the pH of the medium could be protonated. If the reductive potential of the medium is increased superoxide anion radical could transform into hydrogen peroxide, which, in the presence of labile metals, could result in the most active hydroxyl radical that, when it receives an electron, could be detoxified via water formation.

Oxidative stress is the imbalance between the production of reactive, mainly oxygen, species and the ability of the tissue or organism to detoxify them in favor of the first process [1]. Radicals such as superoxide anion radical, hydroxyl radical or singlet oxygen, and other reactive species, such as peroxynitrite and hydrogen peroxide, are generated as metabolic products by biological systems during physiological and pathological processes, such as arachidonic acid metabolism and immune responses. However, their main initiation process is cellular respiration via oxidative phosphorylation or the accumulation of reducing agents such as NADPH and NADPH [2,3]. In Figure 1, the reduction stages of molecular oxygen, the intermediate products superoxide anion radical, hydrogen peroxide and hydroxyl radical, as well as the implication of labile iron in this process are shown.

At a relatively low concentration, radicals play beneficial roles. They adjust various immune pathways, having a key regulatory role in signaling pathways of cells from fibroblasts to endothelial and muscle cells. However, the harmful effects of oxidative stress can affect all the main structures leading to protein, DNA and lipid oxidation, with concomitant impairment of the cell by necrosis and apoptosis (according to the extent of the damage) [4]. Thus, oxidative stress is implicated in conditions such as obesity, cardiovascular diseases, neurodegenerative conditions and cancer and is interrelated with inflammatory processes [5], while normalization of the level of oxidation could improve diabetes and metabolic syndrome [6,7].

Vitamins are vital for life and their lack or over-consumption can cause detrimental effects for the human body [8]. However, their effects derived from their potential antioxidant behavior have not been extensively analyzed yet, especially for all of them together. In view of all the above, in this review we address the influence of oxidative stress on conditions such as diabetes, metabolic syndrome and atherosclerosis via shedding light on the interrelation between oxidative stress and inflammation and emphasizing the potent effects of vitamins on these processes and especially on oxidative load manipulation.

## 2. Cell Degeneration and Oxidative Stress

The manner in which the cell will react to the effects of oxidative stress depends on many factors. At first, cell response depends on the intensity and duration of the stimulus, since high intensity of oxidative stress, caused by either chemicals or radiation, leads to uncontrolled cell death (necrosis) and cell lysis, while oxidative load of reduced intensity and intermittently applied can make the body resistant, activating the cell defense and its antioxidant reserves [1]. Another factor that affects the actions of oxidative stress on the cell is the cell phase. Thus, completely differentiated cells undergo apoptosis or necrosis if they are not capable of damage repair. In mitogenic cells, oxidative load of moderate intensity may initially prevail, inducing cell growth [9], while higher intensity of oxidative stress leads to necrosis or apoptosis [10].

## 3. Inflammation and Oxidative Stress

Inflammation is defined as the local response of the tissues to any damage. In particular, it is the response of the tissues to all kinds of injury in order to attract immune system molecules and cells to the site of damage [11]. The causes of inflammation are various and heterogeneous. In essence, inflammation appears in response to stimuli, as the organism’s first line of defense in offences caused by germs, heat, radiation, mechanical and chemical agents [12]. In the beginning, granulocytes and especially neutrophils phagocytize and neutralize the inflammatory factor. The process of termination of inflammation is then initiated, mainly through programmed cell death—apoptosis [13]—and non-inflammatory phagocytosis by macrophages derived from monocytes that arrive at the site of inflammation after neutrophil influx [14]. The latter are removed either by in situ apoptosis (very little, less than 10%) [15] or by the lymphatic system [16].

The two main categories of enzymes implicated in the inflammation are cyclooxygenases 1 and 2 (COX-1 and COX-2) and lipoxygenases (LOXs). COX consists one of the proteins of Prostaglandin H synthase (an enzyme that contains two active sites) [17]. A tyrosine residue located between the two active centers has been shown to be oxidized, by the corresponding heme, to phenoxy radical and is then ready to receive a hydrogen atom from the arachidonic acid substrate. The cyclooxygenation reaction is carried out by hydrogen atom abstraction via two mechanisms; (1) by radical transfer, hydrogen release and intramolecular cyclization and formation of a cycloperoxy derivative, or (2) by the addition of an oxygen molecule [18]. The formed radical successively takes up two molecules of O_2_ leading to the formation of bicyclic hydroperoxide (PGG2) and finally reduced prostaglandin H2 (PGH2).

By the action of lipoxygenase, the release of a bisallyl hydrogen results in the formation of a lipid alkyl radical (L^•^). Some of the formed alkyl radicals can escape from this enzymatic system and are able to react with O_2_ to form lipoperoxy radicals (LOO^•^) [19]. In addition, the body’s antioxidant defense systems, such as glutathione, are depleted, which seems to cause oxidative stress. The most important inflammatory lipoxygenases are 12-LOX and 5-LOX. However, 5-LOX has attracted more attention due to its involvement in the synthesis of leukotrienes when induced by any inflammatory agent [20]. Arachidonic acid, via the lipoxygenase pathway, is converted to eicosatrienoic acid and leukotrienes.

During inflammation, mast cells and leukocytes are attracted to the site of lysis, leading to a “respiratory burst” (so called due to oxygen consumption carried out through NADPH oxidases), and this is the main element for the antibacterial capacity of phagocytes [21,22] and increased release and accumulation of free radicals at the site of injury [23,24]. Inflammatory cells also produce soluble mediators, such as arachidonic acid metabolites, cytokines and chemokines, which act by attracting other inflammatory cells to the site of inflammation and producing additional free radicals [25].

These mediators can activate signal transduction, as well as induce changes in transcription factors, such as nuclear factor-κB (NF-κB), transcription-activating factor 3 (STAT3) and activator protein-1 (AP-1). Oxidative-load-induced inflammation has been shown to lead to induction of cyclooxygenase 2, an increase in the inducible form of nitric oxide synthase (iNOS), as well as an increase in inflammatory cytokines, such as tumor necrosis factor-α (TNF-α) and interleukin-1 (IL-1) and chemokines, such as the chemokine receptor 4 (CXCR4), in addition to changes caused by the expression of specific microRNAs [26,27,28]. The foregoing lead to the finding that the administration of lipopolysaccharide (LPS, a molecule composed of a lipid skeleton at one end and a glucan skeleton at the other end and that elicits an intense immune response in the tissue and subsequently the body in which it induces TNF-α production) causes the increase in reactive oxygen species (ROS) and the activation of NF-κB [29].

NF-κB has been found to decrease ROS production mainly through induction of the ferritin heavy chain (FHC) genes and superoxide anion radical dismutase-2 (SOD2) [30,31]. In the first case there is the reduction of ROS by blocking the circulation of excess iron and in the second the disproportionation of superoxide anion radical. In contrast, ROS increase NF-κB activation. The mechanism has not been fully established, but it is believed to be through activation of TNF-α and interleukin-1 [32], whereas administration of antioxidants reduces its activation, regardless of which stimulant is used [33]. Such antioxidant inhibitors are molecules containing thiol groups or phenolic hydroxyls, such as L-cysteine, *N*-acetyl-cysteine, green tea polyphenols and vitamin E [11].

**Figure 2:**
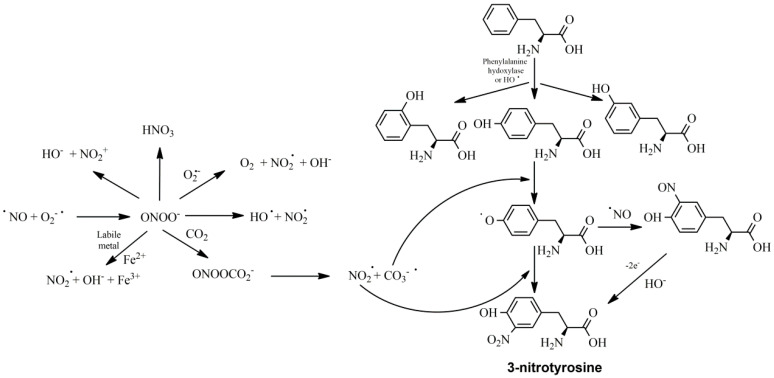
Nitrogen monoxide oxidation and the implication of the resulting reactive oxygen and nitrogen species in the oxidation of tyrosine residues in proteins. A wide array of reactive species can be derived by peroxynitrite and the highly reactive hydroxyl radical can be inserted in many places of the phenyl ring of phenylalanine, leading to successive oxidation and nitrosation processes.

Membrane phospholipids are hydrolyzed by phospholipase A2 (PLA2), releasing arachidonic acid to be metabolized by COX and LOX. Curcumin has been found to inhibit PLA2 and COX-2, as well as LOX. Although it inhibits the catalytic region of 5-LOX directly, it also inhibits PLA2 by preventing its phosphorylation and COX-2 by inhibiting its translation [34]. NF-κB enhances the translation of the COX-2 gene as well as other inflammatory genes, such as the inducible NO synthase (iNOS). In inflammatory cells, such as macrophages, iNOS catalyzes the synthesis of nitric oxide, which in turn reacts with a superoxide anion radical to form peroxynitrite. The latter, due to its high reactivity, can modify protein and DNA structures, such as tyrosine residues (Figure 2). Thus, curcumin has been found to inhibit NF-κB-dependent translation and induction of COX-2 and iNOS in animal and cell line studies [35,36] whilst the administration of a combined antioxidant structure with a nitrogen monoxide (NO)-donating group has shown increased anti-inflammatory potency [37].

## 4. Oxidative Stress, Metabolic Diseases and Cardiovascular Disorders

A high dietary fat intake leads, among others, to insulin resistance for all muscles and especially skeletal muscles, suggesting that it is an important factor in type 2 diabetes and cardiovascular disorders (CVDs) [38,39]. Mitochondrial, secondarily triggered, disorder and oxidative stress have been implicated in these conditions [40]. There is evidence that increased fat intake is responsible for increasing the oxidative load of the cell, especially of H_2_O_2_ produced by the mitochondria, thus reducing the antioxidant regulatory capacity of the cell [41]. Mice receiving antioxidants or transgenic amplification of antioxidant enzymes such as catalase have also been shown to reduce the symptoms of insulin resistance and the production of hydrogen peroxide, even if they receive increased amounts of fat [7].

**Figure 3:**
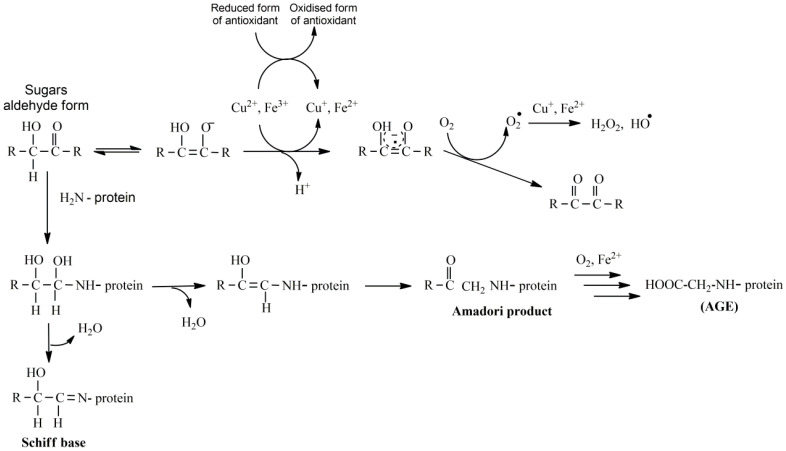
The oxidation process of sugars and the mechanism of formation of the advanced glycation end products. The central pro-oxidant role of the antioxidants in the process of the reduction of transition labile free metal ions is depicted, together with the increase in the production of reactive oxygen species due to the presence of these metals.

The genesis of free radicals (especially O_2_^.−^), from damaged or malfunctioning mitochondria, has been implicated as a trigger for the development of diabetes and its complications [42]. Thus, reduction of mitochondrial free radicals is considered as a goal in reducing the complications of renal dysfunction caused by diabetes [6,43]. Normally, glucose is exploited for energy production while a small part of it is normally metabolized by the polyol pathway. The amplification of the latter pathway in case of diabetes reduces NADPH that is necessary for the reduction of antioxidants in the body (e.g., glutathione). Instead, the concentration of NADH increases, entering the respiratory chain and acting as an electron donor for the production of active oxygen intermediates [44]. Finally, after oxidation of glucose, free aldehyde groups are formed, which react with other amino groups, producing, after many modifications, products not similar to the original (advanced glycation end products—AGEs), partially or completely dysfunctional molecules [45,46] (Figure 3).

The toxicity of AGEs is related to diseases such as cancer, triggering phenomena such as inflammation (macrophage attraction and chemotaxis) and oxidative stress. Yet, another reason intermediates and end products of glycation cause oxidative stress is that, in order to be catabolized or converted into inactive derivatives, they consume endogenous antioxidants, most notably glutathione, which also participates as a catalyst in the conversion reaction of glyoxal in 2-hydroxycarboxylic acid (first oxidized by glyoxylase I and then reduced by glyoxylase II) [47,48]. Thus, during oxidative stress, created in diabetes, the process of regeneration of antioxidant mechanisms begins to be suppressed, indicating that adequate supply of the body with antioxidant derivatives is extremely important.

While adipose tissue represents an important source of free radicals, oxidative stress can also be present in many other tissues, such as the brain, vessel walls and tumors, and events that contribute to hypertension and cancer [1]. Oxidative stress has been implicated in a variety of conditions through a variety of mechanisms, such as pancreatic collapse in diabetes, and obesity is linked to oxidative stress [49]. Obesity, diabetes and the symptoms of the metabolic syndrome, therefore, can help the development of such diseases through oxidative stress and other processes involved [50].

An abnormal increase in malondialdehyde (MDA) levels, as well as low antioxidant potential, has been observed in the liver of hypercholesterolemic mice [51]. Hypercholesterolemia induced the increase in plasma concentrations of enzymes characteristic of liver dysfunction (such as aspartic transaminase—AST, alanine transaminase—ALT and alkaline phosphatase—ALP) together with increased liver cholesterol and altered fatty acid concentrations [52]. Oxidative stress is increased in metabolic syndrome and has been shown to be associated with atherosclerosis. The correlation between the two phenomena is also evidenced by the fact that the ratio of plasma vitamin E concentration to cholesterol was significantly reduced in metabolic syndrome [53].

The intra-thoracic and intra-abdominal adipose tissues are metabolically active and are a source of humoral and cellular inflammation in obese individuals [54]. In addition to atheromatosis, the role of cellular inflammation is involved in other cardiovascular and metabolic events such as hypertension and insulin resistance [55,56]. Therefore, the vicious cycle of adverse health events during hyperlipidemia and metabolic syndrome is understandable via a combination of oxidative and biological stress and through the involvement of inflammatory responses in both of them.

Low-density lipoprotein (LDL) particles have been implicated in atherosclerosis, and, due to their large surface area and high cholesterol concentration, they become more prone to modifications such as oxidation (oxLDL) as well as glycosylation via oxidized sugars, which lead to the final formation of Schiff bases [57]. This transformed LDL does not react with LDL receptors (resulting in reduced lipid uptake and storage by adipose tissue and the subsequent inhibition of lipolysis) but with macrophage scavenger receptors. Thus, LDL becomes more accessible to macrophages [58,59]. Furthermore, with the use of antibodies against AGEs it was shown that glycated LDL is the part that undergoes the most oxidative procedures, leading to the production of oxLDL, demonstrating the cycle of their interaction [60]. LDL is one of the main molecules that reduces the levels of nitric oxide via the oxLDL reaction with NO and the formation of peroxynitrite. In addition, oxLDL stimulates the production of endothelin-1, which acts as a vasoconstrictor [61], a mechanism that is also carried out through catecholamines (e.g., norepinephrine). The catechol ring can easily react with reactive oxygen and nitrogen species, leading to the subsequent oxidation of the ring to quinone and further intensifying the oxidative load.

## 5. Vitamin E and the Potential of Vitamin C

The vitamin E family consists of molecules which contain a chromane ring attached to a 16-carbon atom phytyl side chain. Four tocopherols and four tocotrienols are included in vitamin E. α-Tocopherol is the predominant form of vitamin E. Natural tocopherols have an *R* configuration at all asymmetric centers of the molecule (three in tocopherols with an *RRR* configuration and one in tocotrienols with an *R* configuration) [62]. The metabolism of the derivatives of this family differs. Thus, α-tocopherol binds selectively to the α-tocopherol transport protein (α-TTP) and, with the interference of ATP-binding transporter A1 (ABCA1), α-tocopherol is incorporated into lipoproteins and transported to various tissues via circulation. Unlike α-tocopherol, protected by α-TTP, the remaining forms of vitamin E are largely catabolized in the liver by omega-hydroxylation by cytochrome P450 (CYP4F2) followed by β-oxidation of the phytyl chain to form hydroxy and carboxy derivatives of the initial molecules [63].

The effects of vitamin E are various and most of them widely known [64]. It has immunoregulatory (modifies the expression of transcription factors as well as the activation of macrophages) and antiplatelet properties (inhibits platelet adhesion, which is probably due to the regeneration of the oxidized form of vitamin K) [65,66]. It also possesses indirect anti-inflammatory and antistress properties, in addition to its importance in fetal development [67]. Furthermore, some of its actions are related to the direct binding of certain metabolites, e.g., the 13’-COOH derivative of δ-tocopherol is an inhibitor of COX-1 and COX-2 through competition with arachidonic acid for the active site of the enzyme. However, most of these properties are mainly due to its action as a radical scavenger. All forms of vitamin E are possible antioxidants, as they can bind lipid peroxide radicals, giving a hydrogen atom from the phenolic group of chromane. Trienols, and in particular α-tocotrienol, are considered better antioxidants than α-tocopherol due to the better distribution of tocotrienol in the phospholipid bilayer, thus giving optimal access to the space in which the lipid peroxides are generated [68].

Binding of peroxynitrites, regardless of how they originate (e.g., by induction with lipopolysaccharide), can be accomplished by vitamin E and thereby indirectly reduces the action of cyclooxygenases [66]. Accordingly, vitamin E reduces the production of IL-6 by suppressing the activation of NF-κB, caused by the administration of lipopolysaccharide [69]. In addition, vitamin E and its metabolites possess anti-inflammatory properties. It is noteworthy that γ-tocopherol is more active than other forms of vitamin E in inhibiting the production of eotaxin-3 by IL-13, apparently preventing the phosphorylation of the transcription factor STAT6 and binding to DNA, respectively, in the human lung epithelium. In neutrophils, vitamin E suppresses the entry of calcium, stimulated by sphingosine phosphate 1, and the signaling cascade it creates [62]. These signals include the activation of phospholipases that are responsible for initiating the production of eicosanoids and the disruption of the integrity of biological membranes and the cell.

**Figure 4:**
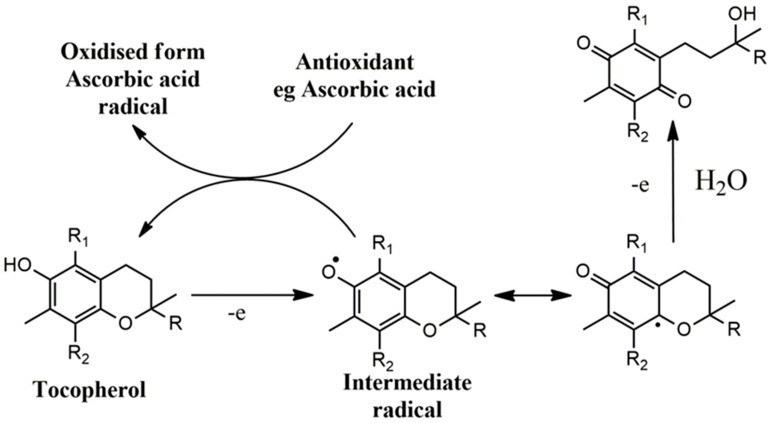
The oxidation mechanism of tocopherols and the regenerating activity of endogenous or exogenous antioxidants. When the intermediate radical of tocopherol is not reduced, it may result in its full oxidation and the formation of the final quinone structure, with a decrease in the levels of tocopherol.

However, vitamin E and its derivatives could act pro-oxidantly in the case of a lack of regenerating molecules. This is derived from the relatively low oxidative potential of the intermediate radical formed during the initial electron abstraction from the chromanol ring, whilst, in the presence of other antioxidants or the intramolecular insertion of antioxidant structures, this effect can be inhibited by restoring the vitamin E analog to its initial state (Figure 4) [70,71,72]. However, if there is a second electron abstraction, the final formation of a quinone structure, after hydrolysis, is irreversible. Additionally, vitamin E interferes with the activity of other vitamins like vitamins K1 and K2 at various levels involving truncation of the latter, cytochrome P450 antagonism for beta-oxidation and induction of vitamin K forms’ metabolism [73]. Although there is evidence of pleiotropic activities and antioxidant effects of vitamin E, the lack of statistical significance or clinical importance in important cardiovascular events and in observational epidemiological studies accentuates the need for more information. The failure of vitamin E therapy may be linked to factors that concern the poor method of the studies, such as the duration, the usage of suboptimal doses, the administration and fasting mode, the patients’ compliance and the lack of concurrent use of vitamin C for boosting the effects of vitamin E [74,75].

Vitamin C is an important compound for the body, since it directly scavenges ROS, and especially peroxyl radicals, or indirectly regenerates lipid soluble molecules, ameliorating lipid, protein and DNA oxidative damage [76]. However, like vitamin E, the same pro-oxidant effect can take place in the case of vitamin C, since high doses of vitamin C induce cellular ROS formation and mitochondrial impairment, decreasing membrane potential [77]. This may, in part, be attributed to the formation of the intermediate ascorbate radicals and hydrogen peroxide or the presence of labile metals, e.g., Fe^3+^, since vitamin C reductive activities give the potential to reduced iron to participate in the Fenton reaction. Apart from the direct antioxidant and pro-oxidant effects, vitamin C has been shown to decrease inflammatory apoptotic conditions such as cytochrome C release, the activation of caspase-9 and the stabilization of tetrahydrobiopterin, leading to increased production of NO and inhibition of NOS decoupling, with effects on endothelial survival rate and smooth muscle cell proliferation and differentiation [78,79]. Vitamin C or E alone or co-supplemented in male patients with type 2 diabetes mellitus has shown to improve fasting blood glucose, glycated hemoglobin (HbA1c), insulin resistance and the lipidemic profile, with a parallel increase in the antioxidant status of the body (statistically significant increase in reduced glutathione levels), contributing to the increase in the endogenous antioxidants and ameliorating the diabetic pathogenesis and complications [80]. These effects were accompanied by improvement of markers concerning the liver function (alanine transaminase (ALT) and aspartate transaminase (AST)) and urea levels and intensification of the effects of metformin on cholesterol, triglycerides and low-density lipoprotein (LDL) and high-density lipoprotein (HDL) cholesterol, together with diabetes amelioration. These results of vitamin C are in accordance with a meta-analysis of randomized controlled trials (RCTs), with the exception of the insignificant effects of vitamin C on serum values of homeostasis model assessment of insulin resistance [81]. However, according to the latter, high doses (≥1000 mg/d) and long-term treatment are needed for the potential amelioration of the hyperglycemic profile, an event that dictates the need for more research, clinical data and RCTs.

## 6. Vitamin A

Vitamin A and its metabolites have a pivotal role in the preservation of homeostasis. The suppression of their signaling cascade has been shown to provoke liver cancer and oxidative stress, and this may, in part, be mediated via the suppression of the expression of thioredoxin-interacting protein, giving thioredoxin the ability to improve the free thiol/disulfide portion [82]. Nuclear transcriptional suppressors may be recruited by the heterodimers of retinoic acid receptors and retinoid X receptors, repressing target gene expression [83]. Additionally, 9-*cis* retinoic acid supplementation has led to up-regulation of the antioxidant-related genes in buffalo oocytes, but in a dose-dependent manner, since in high concentrations these actions seem to be reversed [84]. This is in relation to the appropriate mitochondrial function and diminished electron leakage leading to decreased ROS formation and cell apoptosis at low and not at higher concentrations [85]. Additionally, *all-trans*-retinoic acid (AtRa) seems to have an anabolic effect on somatic cell metabolism. It increases lipase activity and decreases triglyceride and glucose levels, with a further inductive effect on glucose-6-phosphate dehydrogenase (G6PD). Thus, the survival rate of the cells is increased potently via AtRa’s superoxide dismutase effect and the ability to regenerate glutathione by G6PD activity [86].

Vitamin A is essential for the development and function of β cells, whilst its deficiency may result in hyperglycemia and diminished glucose-stimulated insulin secretion [87]. Since vitamin A is essential for immune system regulation, its reduced levels could affect immunity and the onset of type 1 diabetes (T1DM), and despite the fact that human studies are scarce, low serum circulating levels of this vitamin in T1DM patients are indicated [88]. Thus, the administration of all-*trans* retinoic acid seems to delay the onset of diabetes and the destruction of β cells with an increase in the number of immunosuppressive T-regulatory cells [88]. All-*trans* retinoic acid has also been shown to increase glucose-stimulated insulin secretion and cyclic adenosine monophosphate (cAMP) levels in mouse islets, an effect that is, at least partly, related to the agonism of the orphan G-protein coupled receptor C5C (GPRC5C) [89]. Vitamin A has also been linked with reduced risk of metabolic syndrome [90], with the results being contradictory to the findings of other studies [88].

## 7. Thiamine, Riboflavin

**Figure 5:**
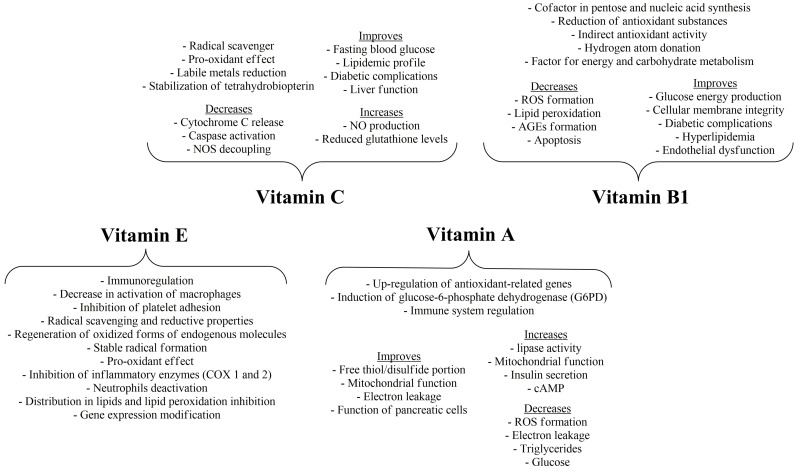
Activities of vitamins E, C, A and B1.

Thiamine (vitamin B1) improves glucose energy production and is involved as a co-factor in pentose and nucleic acid synthesis, together with reducing antioxidant substances [91] (Figure 5). Thus, a lack of thiamine has been related to apoptosis and neurodegeneration due to energy production issues, excessive ROS formation, cellular membrane leakage, caspase-3 activation and translocation of amyloid precursor protein-C, thus provoking mitochondrial dysfunction with increased glycolysis and lactate dehydrogenase accumulation, free radical formation and subsequently glutathione depletion [92]. Genetic changes in the transportation of thiamine may result in systemic disorders, whilst thiamine treatment has been linked to improvement of nerve conduction velocity and complications such as neuropathic symptoms, diabetic retinopathy, microalbuminuria, hyperlipidemia and AGE formation in plasma [93]. Furthermore, the lipophilic thiamine derivative benfotiamine seems to reverse the endothelial dysfunction and the oxidative conditions accompanying type 2 diabetes and the apoptosis in high-glucose-cultured vascular cells [92,94]. These effects accentuate the indirect antioxidant ability of thiamine. However, it has been documented that it is able to inhibit lipid peroxidation and oleic acid oxidation in vitro, since it is oxidized to thiochrome and thiamine disulfide by transferring two hydrogen atoms from the amino group of the molecule and one hydrogen by the thiazole ring [95]. Since thiamine is an important factor for energy and carbohydrate metabolism (co-factor for pyruvate dehydrogenase and alpha-ketoglutarate dehydrogenase), its deficiency has been linked to neurological and cardiovascular abnormalities, with patients suffering from heart failure having statistically significantly more prevalent deficiency [96,97,98].

Riboflavin (vitamin B2) is crucial for cellular and drug metabolism, potentially via its implication in iron metabolism, since, via the reduction of riboflavin by NADPH, the necessary electron transfer in cytochrome P450 takes place [99]. Riboflavin acts directly as a co-enzyme in flavin adenine dinucleotide (FAD) and flavin mononucleotide (FMN), since FAD is essential for glutathione reduction via glutathione reductase, and riboflavin deficiency may increase lipid peroxidation. Administration of riboflavin seems to reverse this effect, with reduced levels of malondialdehyde and oxidized proteins [100,101]. However, these effects may be independent of the glutathione redox cycle, since it is proposed that the antimutagenic effects of riboflavin may be derived from the direct scavenging of ROS and RNS, e.g., hydroperoxides, produced by mutagens, or by the reinforcing effect of riboflavin on other antioxidant vitamins, e.g., vitamin C [102]. In addition, in the ferric-reducing effects the flavin reductase activity of methaemoglobin reductase may be included, leading to protective effects against reperfusion oxidative injury [103]. Riboflavin has anti-inflammatory and antinociceptive, effects, whilst it can activate glutathione reductase and the regeneration of glutathione (GSH) and prevent neutrophil migration and infiltration via mechanisms implicating NF-κB regulation, since riboflavin could also act as a proteasome inhibitor [104,105].

## 8. Niacin and Pantothenic Acid

Niacin (vitamin B3) comprises nicotinic acid, nicotinamide and various forms, e.g., nicotinamide adenine dinucleotide (NAD) and nicotinamide adenine dinucleotide phosphate (NADP) implicated in energy manipulation from lipids, proteins and carbohydrates [106]. Apart from the well-defined activity of NADPH on glutathione reductase, niacin has been shown to increase the levels of the most important antioxidant paths in the human body, e.g., catalase, glutathione peroxidase and superoxide dismutase, decreasing lipid peroxidation [107]. On the contrary, a niacin-deficient diet increased lipid peroxidation biomarkers, an effect that seemed to be reversed after the administration of the vitamin, with a subsequent increase in vitamin E and glutathione levels [108]. In another study, niacin decreased the oxidative insult of hepatocytes and fat accumulation, with a parallel decrease in NADPH oxidase activity and interleukin-8 levels [109]. Niacin may be of assistance in combination with exercise since it increases paraoxonase-1 activity and concentration without any increase in lipid oxidation markers [110]. Niacin may have cardiac-protective effects due to its lipid-lowering capacity, however, apart from this, the free fatty acid uptake and utilization and the preserved ratio of NADH/NAD may assist towards improved cell respiration and ATP production [111]. A three-month treatment with niacin in a controlled, double-blinded, single-center trial gave a significant change in the HbA1c levels with secondarily lower results in fasting blood glucose and lipoprotein concentrations [112]. Additionally, reduction in fasting blood glucose and recovery of the liver and kidney tissues has been observed in an alloxan-induced diabetes model, with a notable improvement of oxidative and DNA damage markers [113]. However, its effects on cardiovascular protection are questioned, since in high doses it is linked to vessel inflammation and increases in cardiovascular disease risk [114].

Like the abovementioned compounds, pantothenic acid (vitamin B5) has a central role in energy metabolism via its incorporation into co-enzyme A, implicated in glucose, lipid and amino acid reactions and biosynthesis [115]. The incubation of Ehrlich ascites tumor cells with pantothenic acid led to a substantial reduction of energy metabolism and leakiness, but only in the case of pre-incubation, indicating the indirect metabolic effects of the vitamin on cellular metabolism and antioxidant status improvement in comparison to direct radical scavenging ability [116,117]. Thus, increased glutathione content and GSH/GSSG ratio were observed [116]. These beneficial results were also obtained in vivo using γ-irradiation exposure of adult rats, preventing liver oxidative damage, as well as glutathione, catalase, glutathione reductase and peroxidase decrement [116]. Furthermore, pantothenic acid may have an antioxidant effect in inflammatory processes, since it is inversely related to *C*-reactive protein concentration in healthy middle-aged or older people [118]. Lower baseline levels of pantothenic acid have been shown in elderly men with diabetes mellitus and cognitive decline [119], and pantethenine (a derivative of vitamin B5) treatment showed a decrease in lipidemic markers such as LDL, HDL and total cholesterol levels [120]. Decreased pantothenic acid levels have also been associated with increased risk of coronary heart disease, with the supplementation of the vitamin significantly decreasing the risk up to a threshold, that could not be overcome in concentrations of pantothenic acid ≥ 44.0 ng/mL [121].

## 9. Vitamin B6, Biotin, Folic Acid and Vitamin B12

In the trans-sulfuration process, vitamin B6 is highly involved in two vitamin-B6-dependent reactions resulting in proportionally half of the cysteine used for glutathione synthesis [122]. In addition, vitamin B6 assists in the insertion of toxic selenides into organic proteins, such as glutathione peroxidase, resulting in an antioxidant-detoxifying molecule in preference to the excretion of selenide via methylation reactions [123]. These effects render vitamin B6 important for the antioxidant status of the body and even for the preservation of its one-carbon pool. Apart from these effects, pyridoxine has been shown to act as a hydroxyl radical scavenger preventing lipid peroxidation in monocytes, possibly via alterations in mitochondrial function [124]. This lipid-preserving activity is accompanied with atherogenic processes that take place during the deficiency of vitamin B6. The direct radical-scavenging action of vitamin B6 is not well documented; however, there is a theoretical view that renders vitamin B6 capable of binding hydroxyl radicals on the pyridoxine aromatic ring or abstracting hydrogen atoms from the non-aromatic subgroups [125,126]. Pyridoxine has been shown to decrease the risk of islet autoimmunity with a decrease in multiple autoantibodies, in T1DM-susceptible children, in a prospective observational birth cohort study [127]. Its deficiency has been associated with reduced nerve conduction velocity, with an inverse relationship between the levels of the vitamin and those of fasting blood sugar and HbA1c, glucose tolerance [128] an increased risk of cardiovascular disease [129].

**Figure 6:**
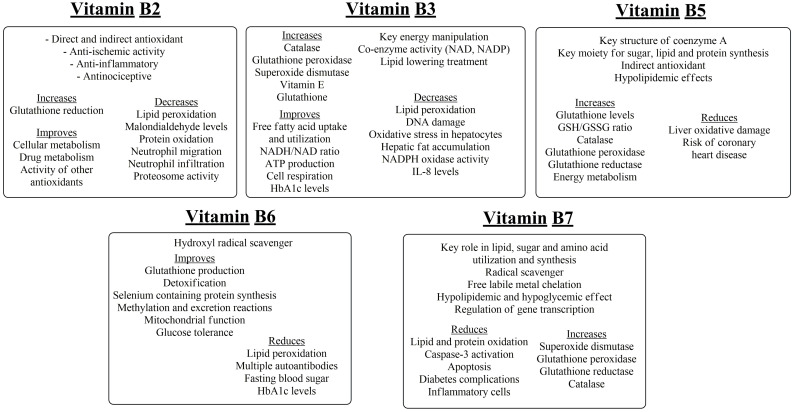
Activities of vitamins B2, B3, B5, B6 and B7.

Biotin (vitamin B7) is a water-soluble vitamin involved in many metabolic processes in mammals and other species, with its main relation being to lipid, sugar and amino acid utilization by tissues [130]. However, biotin has been shown to influence the scavenging of superoxide anion and hydroxyl radicals in fish muscle. This effect may be related to an indirect increase in copper chelation by biotin supplementation and the enhanced activities of superoxide dismutase, glutathione peroxidase, glutathione reductase and catalase [131] (Figure 6). These findings are in accordance with those of Sghaier et al. [132] that biotin supplementation leads to the attenuation of the oxidative 7β-hydroxycholesterol, together with protein and various other lipid oxidation products. Thus, these effects led to normalized lipid synthesis and diminished apoptosis, preventing mitochondrial radical formation and caspase-3 activation. These activities of biotin are interrelated with hypolipidemia or hypoglycemia and diabetes-complication-reducing effects and are associated with transcriptional changes in key metabolic organs [133]. Biotin supplementation could offer a decrease in fasting blood glucose, total cholesterol and triglyceride levels in T2DM patients [134] and improvement of glycemic control and insulin sensitivity in T1DM patients with an increase in the expression of the hepatic glucokinase gene [135]. Biotin has been shown to reduce inflammatory cells, such as giant macrophages, in the kidney and leukocyte infiltration in the liver [133]. These actions may depend on NO concentrations and decreased immune reaction responses [136]. Furthermore, insufficient amounts of biotin have been linked to elongation and desaturation of fatty acids, with potentially negative impacts for dyslipidemic and cardiovascular events [137].

Vitamins B12 and folic acid (vitamin B9), as well as vitamin B6, are the main co-factors in the methylation and trans-sulfuration processes that lead to the formation of methionine via the subsequent homocysteine methylation (homocysteine oxidation is implicated in ROS production) and cysteine formation, respectively [138]. It has been shown that the supplementation of vitamin B12 and folate enhances GSH levels in colonic tissue. The most potent mechanism is phospholipid methylation, counteracting the oxidatively stressful conditions induced by azomethane administration [139]. Their supplementation reduced the expression of B cell lymphoma protein (Bcl-2 protein), balancing their antiapoptotic signals and chemotherapy resistance. Furthermore, they reduce 8-hydroxy-2′-deoxyguanosine levels in cerebrospinal fluid, with methionine synthase inhibition partly related to this effect [140].

Vitamin B12 in physiological concentrations may decrease superoxide radicals’ level in human aortic endothelial cells and in retinal ganglion cells, resulting in cell survival, via a direct radical-scavenging mechanism, indirect homocysteine reduction and preservation of glutathione potential [141]. The antioxidant effects of vitamin B12 may be interrelated with its anti-inflammatory properties, since, in vitamin-B12-deficient patients, increased levels of IL-1 and TNF-α have been observed in comparison to non-deficient patients [141,142], suggesting the cytokine-modulating properties of vitamin B12. On the other hand, oxidative stress may reduce vitamin B12 uptake via the formation of AGEs that contribute to radical formation, creating a feedback cycle of subclinical vitamin B12 levels and oxidative stress [143].

Folic acid and its various reduced and methylated forms may exert their antioxidant effects, apart from homocysteine-adjusting properties, via nitric oxide enhancement, leading to improved vascular tone and reduced NO synthetase dissociation, while a direct effect of the reduced forms of folate may offer protection against LDL oxidation [144]. Folic acid may scavenge hydroxyl and thiol radicals, offering antioxidant activity and protection against lipid peroxidation from thiyl radical attack [145].

Folic acid administration was related to 10% and 4% lower risk for stroke and CVD incidence, respectively [146]. Vitamin B12 supplementation (500 mcg/day), in a randomized, multi-arm, open-label clinical trial of patients with T2DM, resulted in a significant decrease in HbA1c, whilst co-supplementation with folic acid gave significant improvement of insulin levels in plasma and of insulin resistance, with a parallel increase in adiponectin levels, which may further indicate the anti-inflammatory and insulin-sensitizing potential of the treatment [147]. The anti-inflammatory dynamic of vitamin B12 was also shown in another study of patients with high cardiovascular risk, where higher vitamin B12 levels were correlated with a decrease in IL-6 and *C*-reactive protein (CRP) levels [148]. The same inverse relationship between these markers and vitamin B12 was also recorded in the same study [148] in naturally aged mice. However, an increased serum vitamin B12 concentration (>600 pmol/L) has been linked with increased all-cause mortality and CVD mortality [149], accentuating the need for timely and effective management of balanced vitamin B12 and folic acid levels in clinical practice.

## 10. Vitamin D

Vitamin D comprises two lipid-soluble forms, vitamins D3 (cholecalciferol) and D2 (ergocalciferol). The latter is mainly ingested through the diet while the former is synthesized via sunlight exposure and then is converted to its active metabolite via two subsequent hydroxylations, one in the liver (25-hydroxyergocalciferol) and the other in the kidneys (1,25-dihydroxycholecalciferol, calcitriol) [150]. Apart from the expected actions on bone metabolism and the transfer and distribution of calcium and magnesium in the body, it has a wide array of activities in a genomic and non-genomic manner [151]. The genomic effects are mediated via the binding of vitamin D to vitamin D receptor (VDR) with gene- and cell-specific activities, whilst in the non-genomic, very rapid phase, there is a signal transduction, leading to direct activation of various factors [152].

Vitamin D acts as a regulator of the renin–angiotensin system and an inducer of insulin secretion [153]. Furthermore, vitamin D offers cell-modulatory effects via NF-κB-inhibitory effects, diminishing oxidative and cellular inflammatory responses [154] and offering anticancer cell-differentiating effects [155]. The effect of a low concentration of calcitriol is associated with oxygen and nitrogen reactive species accumulation and endothelial dysfunction [156]. These effects may, in part, be related to the effect of vitamin D on metabolic imbalances, such as metabolic enzymes, implicated in glucose degradation and oxidative phosphorylation [157], or via gene-modulatory effects leading to mitochondrial wellness and effective respiration processes, protecting the cell from the production of excessive ROS and RNS [158].

The antioxidant activity of vitamin D includes the enhanced expression of the indirect antioxidant nuclear factors erythroid-2(Nf-E2)-related factor 2 (Nrf2) and Klotho [159,160], leading to decreased mitochondrial ROS production [161]. Furthermore, Nrf2 interacts with peroxisome proliferator-activated receptor-coactivator 1α (PGC-1α), regulating mitochondrial deacetylase (SIRT3), under the influence of vitamin D and its metabolites [162]. Calcitriol can exert its beneficial effects via regulation of some of the abovementioned antioxidant mechanisms and inflammatory cytokines, offering protection from micronutrient deficiency and harmful conditions, via interrelated adjustment of ROS and inflammatory processes, on the basis of cell signaling pathways [163]. Thus, vitamin D deficiency is associated with necrosis and apoptosis (via increased inflammation and telomere shortening), increased expression of TNF-α and intracellular calcium concentration and is considered an important risk factor for increased mortality, as well as cardiovascular and metabolic dysfunctions [164].

Vitamin D plays a vital role in the production of glutathione, since it regulates the expression of key enzymes (e.g., glutathione reductase and glutamyl transpeptidase) for the synthesis of glutathione [165]. Apart from this, vitamin D up-regulates the expression of glutathione peroxidase and superoxide dismutase and affects the regeneration of glutathione through the increased production of NADPH (by the activation of glucose-6-phosphate dehydrogenase), an essential step for the reduction of glutathione disulfide to the corresponding glutathione [165]. Vitamin D deficiency is also responsible for the decrease in the expression of complex I in the respiratory chain, leading to decoupling of electron transport and decrease in energy production, leading to mitochondrial and subsequent harmful cellular effects and leading to mitochondrial and subsequent harmful cellular effects. Especially in cases of exposure to toxic stimuli and in chronic diseases, disruptive signaling pathways are triggered, such as tyrosine phosphorylation and protein kinase activation [163]. Furthermore, the VDR may offer protection to the endothelium through the inhibition of cholesterol uptake by macrophages, inhibiting cell proliferation, cytokine release and oxidative burst. Vitamin D protects neurons from excitotoxicity by NMDA and reactive oxygen species and offers inhibition of inducible NO synthetase (iNOS) [166,167]. The above mentioned a vicious cycle of inflammatory and oxidative conditions linked partly with diminished vitamin D concentration. Vitamin D3 could decrease advanced oxidation protein and glycated hemoglobin (HbA1c) levels in three months (with higher vitamin D3 doses) and six months of supplementation, respectively, whilst it could be concomitantly administered with antidiabetic drugs, with low incidence of interactions or adverse events [168]. Additionally, randomized control trials have shown glycemic control (serum fasting plasma glucose decrease) and insulin resistance improvement, together with improvement of the T2DM derived complications [169].

## 11. Vitamin K

**Figure 7:**
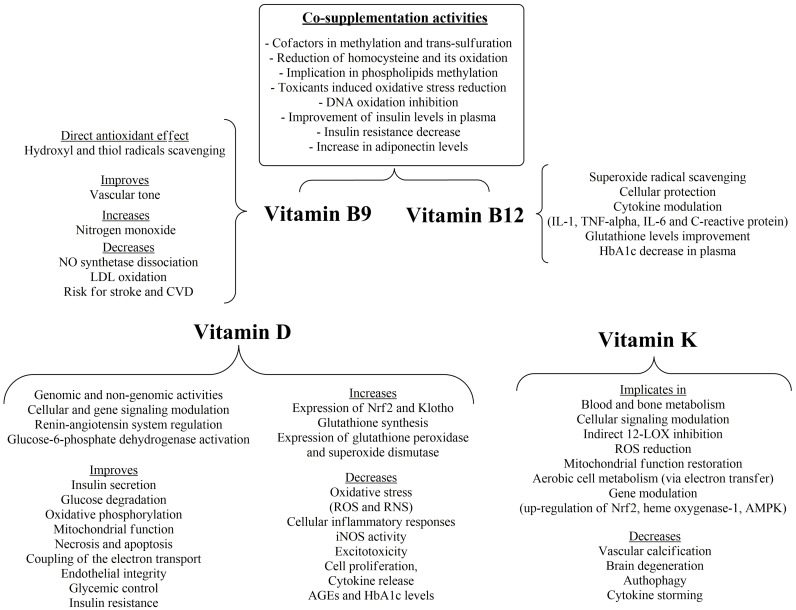
Activities of vitamins B9, B12, D and K.

As far as vitamin K and its derivatives are concerned, they are implicated in, and metabolized by, the γ-glutamylcarboxylase pathway involved in blood and partially in bone metabolism. However, vitamin K has been shown to be metabolized to various non-canonical derivatives and act as a cellular-signaling-modulating factor [170]. Despite its inactivity in direct inhibition of 12-lipoxygenase (12-LOX), vitamin K is implicated in 12-LOX inhibition by interfering in the pathways of its activation (Figure 7). In this effect, the active metabolites of vitamin K may be implicated [171]. The lipoxygenase-inhibitory effect is of high importance to the ROS-inhibitory effect due to the interrelation between the inflammatory and the oxidative effect of 12-LOX. Furthermore, vitamin K, and especially in co-administration with vitamin C, has been shown to restore mitochondrial function, acting as an electron transfer mediator from co-enzyme Q to cytochrome c, thus improving the aerobic cell metabolism [172]. This effect is mediated via the interconversion of vitamin K between quinone and hydroquinone forms through the intermediate semiquinone. This process demands reducing equivalents that may be superoxide radicals, and in this case, it is favorable. However, if this process depletes NADH, NADPH and glutathione, it is unfavorable because it leads to ROS-scavenging inability of the cell. Especially in the presence of free labile metals, e.g., Fe^3+^, the Fenton reaction may take place with the assistance of the vitamin K semiquinone form [173,174]. This implies the indirect antioxidant potential of vitamin K, but mostly its oxidative phosphorylation and redox homeostasis restoration potentiality. Additionally, vitamin K has gene-modulating activity, such as for growth arrest-specific gene 6 (GAS6), which seems to reduce cell death via up-regulation of Nrf2, heme oxygenase-1 and the cellular energy sensor AMP-activated protein kinase (AMPK), improving cellular survival and mitochondrial function [175]. Vitamin K can also activate proteins responsible for reduction of vascular calcification and improve brain degeneration, insulin sensitivity, autophagy and cytokine storming, rendering it a promising tool for diabetic, cardiovascular and thromboembolic events [176].

## 12. Lipoic Acid, Low-Molecular-Weight-Thiol-Containing Compounds, L-Carnitine and Co-Enzyme Q10

*R*-lipoic acid (*R*-5-(1,2-dithiolan-3-yl) pentanoic acid) was discovered by E.E. Snel in 1937, who found that certain bacteria needed a potato extract to grow. This so-called potato growth factor was found to be lipoic acid and was considered as a vitamin until it was discovered to be produced by plants and animals. It is an important compound, since it participates in biological reactions necessary for metabolism, such as its amide derivative with the lysine side amino group, which contributes as a co-factor in mitochondrial enzymes, catalyzing the oxidative decarboxylation of pyruvic acid and the side chain of α-ketoacids [177]. Although the biosynthetic pathway of lipoic acid is not fully documented, it appears to take place in mitochondria from octanoic acid and sulfur-containing compounds [178].

α-Lipoic acid (LA) and its reduced form (dihydrolipoic acid) possess radical-scavenging and metal-chelating activities, the capability of interaction with other antioxidants, amphiphilic nature and good bioavailability and safety, characterizing them as useful antioxidants [179]. LA binds to reactive oxygen species, such as hydroxyl radicals, hypochlorous acid (HOCl) and single-state oxygen. It does not appear to bind hydrogen peroxide or superoxide anion radicals. These actions result from the immediate binding of these active compounds and not from the conversion of LA to the reduced derivative. Due to the free thiol groups, dihydrolipoic acid has the ability to bind with higher affinity, both in number per mole and in radical species, with active forms of oxygen, as well as to complex more strongly with different metals, in comparison with lipoic acid. LA forms stable complexes with Mn^2+^, Cu^2+^ and Zn^2+^, and the complexes are attached to the carboxyl group (i.e., by ionic bonds). Similarly, dihydrolipoic acid forms chiral compounds, in a more powerful chelating manner than LA, with iron (thereby restricting its ability for oxidation) and other metal ions [180].

Lipoic acid has an asymmetric center in its dithiolate ring. It is believed that during the evolution of species, the active site of the proteins and the enzymes associated with it have been shaped in order to fully adapt to the *R*-isomer. However, thioredoxin, an enzyme involved in the regeneration of sulfhydryl groups of proteins and in the redox balance of the cell, shows slight selectivity for the *S*-isomer. The same is considered for glutathione reductase, which uses the *S*-isomer as a substrate [179,181].

Through the decrease in NADH and NADPH, which is directly caused through the reduction of lipoic acid to its dihydro form, but also through its involvement in the metabolism of pyruvate acid via the formation of lipoylCoA, lipoic acid contributes to the treatment and improvement of disease conditions, such as diabetes mellitus. In diabetes, lipolysis and glycolysis increase, leading to an increment in NAD(P)H [181].

The issue of compounds bearing one, two or more thiol groups is of concern to the scientific community. Such compounds are penicillamine, cysteamine, cysteine and its derivatives, dimercaprol, glutathione and various others. These molecules exert their protective action mainly via two pathways. The first concerns the binding of metals, especially heavy metals, through their association with the thiol group or the amine and carboxylic moiety of the molecule. One such example is cadmium, which is known to bind to human albumin as well as transferrin and ferritin [182,183]. However, it is not known yet which of these proteins is responsible for transporting and installing this toxic metal in the respective tissue. What is known, however, is that cysteine (cys) and glutathione (GSH) can displace cadmium from these proteins. Depending on the concentration, binding of cysteine results in either complex Cd (cys)_2_ or Cd (cys)_4_ leading to the final complex being removed from the kidneys or sometimes transported by active out-of-circulation transport and re-introduced even after several hours [183]. Penicillamine, which chelates copper and is used in combination with zinc sulfate in the treatment of Wilson’s disease, belongs to this category of chelating agents [184]. The second pathway induces the synthesis of metallothioneins in cells, which bind copper (again via the thiol groups), keeping it in a reduced form, not allowing it to oxidize, carrying out the Fenton reaction and preventing the vicious oxidation cycle.

Low-molecular-weight-thiol (LMWT)-containing compounds have the additional ability to react with radicals as well as with hypochlorous acid. This reaction is ten times faster at pH 7.4. This further emphasizes the importance of the hypochlorous acid inactivation reaction by thiol compounds in biological systems. Thus, the question that arises is which thiol compounds can achieve the treatment of inflammation, since HOCl is known to be a product of macrophages, during the respiratory burst, at the site of inflammation [185].

**Figure 8:**
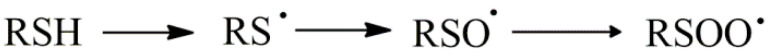
Radical formation from thiols.

Thiols can, by reacting with hydrogen or organic peroxides, but also with peroxynitrites (inactivating them, thus detoxifying the body), be oxidized to compounds such as sulfenic and sulfonic acids, as well as to one-atom sulfur in the form of an oxide. Similarly, with peroxiredoxins, a cysteine residue is converted to sulfenic acid and then reacts with a free thiol to form disulfide. The reaction cycle is terminated after conversion of the disulfide to free thiol by simultaneous oxidation of a cysteine residue of another subunit of the enzyme [186]. Disulfide formation is necessary for the final reduction of oxidized thiols via thioredoxins. Thiols have antioxidant potency, but they might also reinforce oxidative processes via the formation of thiol radicals (Figure 8).

Cysteine has been found to inhibit the oxidation of myoglobin and partial restoration of mitochondrial function was accomplished by *N*-acetyl-cysteine [187,188]. However, due to the complex nature of LMWTs, they possess a variety of properties, which are largely due to the formation of sulfur radicals and cytotoxic reactive oxygen species, resulting in further destruction of cell structures such as DNA and lipids but also in the binding of transition metals [188,189]. It should also be noted that cysteine, among others, is the source of hydrogen sulfide production by the body through two pyridoxal phosphate-dependent enzymes, cystathionine β-synthetase and cystathionine γ-lyase. Hydrogen sulfide has vasodilating properties, possibly acting as a signal transmitter (gasotransmitter) [190]. Hydrogen sulfide can also reduce the swelling and adhesion of leukocytes, inhibit the synthesis of inflammatory cytokines and increase the repair of gastric epithelium, which renders it a good anti-inflammatory gasotransmitter and, in combination with other known anti-inflammatory molecules, it can exert cellular protective properties [191,192].

Despite the fact that L-carnitine is not considered a vitamin, it is an important nutrient acting as a co-factor in β-oxidation of fatty acids and a transfer assistant of acetyl groups through the mitochondrial membrane [193]. L-carnitine has been shown to delay mitochondrial dysfunction in rats and stabilize mitochondrial ROS production [194]. L-carnitine levels were positively correlated with increased levels of catalase, superoxide dismutase and glutathione peroxidase and the decrease in malondialdehyde in patients with coronary artery disease [195]. L-carnitine has shown antioxidant and protective effects in vitro in hydrogen-peroxide-induced cytotoxicity related to the increased activity of catalase and superoxide dismutase, whilst it attenuated the decreased expression of peroxisome proliferator-activated receptor (PPAR)-alpha (PPAR-α) [196]. Co-enzyme Q10 (CoQ10), a vitamin-like, lipid-soluble compound, is related to electron transfer via the respiratory chain, and its reduced levels are correlated with heart, nervous and muscle degeneration or deterioration, whilst an increase in its levels is associated with decrement in vascular dysfunction and cardiovascular events in general, although long-term treatment regimens are needed [197,198,199]. These positive effects derive from the direct improvement of adenosine triphosphate (ATP) and reduced nicotinamide adenine dinucleotide (NADH) form production and the increase in SOD-1 levels, resulting in protection of lipids, blood flow and vessels [200,201]. CoQ10 is also potent as a co-treatment option, since it can reduce the undesired effects of other treatments, such as the rhabdomyolysis caused by statin administration, with parallel improvement of plaque size and vascular dysfunction [202,203].

## 13. Conclusions and Future Perspectives

Mitochondrial dysfunction and metabolic abnormalities are directly and indirectly linked to aging and degenerative processes and could be restricted by nutrient supplementation, playing a pivotal role in co-enzyme boosting and decreased intracellular calcium levels, maintaining autophagy, inflammation and DNA damage at low levels by the reduction of the oxidative insult.

**Figure 9:**
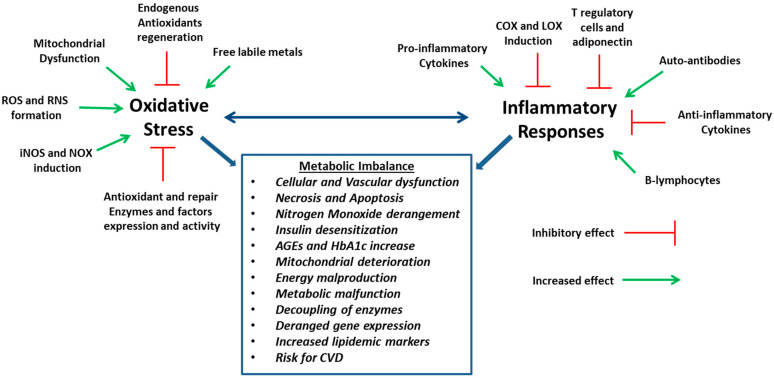
Depiction of the potential implications of vitamins for metabolic abnormalities via their antioxidant and anti-inflammatory potential, intervening in factors influencing the interrelated oxidative stress, inflammatory responses and metabolic imbalance.

Oxidative stress is linked to the progression of cardiovascular disturbances and maladjusted metabolism, diabetes, hyperlipidemia and vascular dysfunction (Figure 9). The nutrients discussed in this review adjust several endocrine, paracrine and autocrine systems or act as co-enzymes. In these processes, the oxidative effect is highly implicated in inflammation and energy metabolism, offering optimal cellular function or dysfunction, according to its level. The rate of oxidative stress is modulated by physical factors, such as the environment, epigenetic pollutants and lifestyle. In this direction, the preservation of the adequate concentration of these vitamins in the long term and in a steady state may be of assistance for the optimal handling of oxidative stress. These effects may derive from the direct or indirect antioxidant and anti-inflammatory characteristics of vitamins and the adjustment in the levels of these parameters. Thus, vitamins may have a more pronounced potential in health and nutrition and the translation of the knowledge of their mechanisms in terms of antioxidant and multi-targeting capacity could lead to health promotion. These effects are essential for the preservation of the body’s homeostasis. However, evidence for optimal tissue, cellular and blood concentration of the vitamins is relatively scarce, and although very low levels of vitamins are known to deteriorate the body’s function, the extent of the negative effects at mildly low levels remains to be concluded. Many topics, such as the supplementation dose and period for each vitamin and condition, co-administration of vitamins with each other or other nutrients and toxicity and systematic or non-systematic accumulation in the body issues, need to be addressed and analyzed in a more thorough manner, in the future, with pre-clinical and clinical studies and evaluation of oxidative, inflammatory, general and specific healthy state markers.
